# Exposing Fraudulent Colleges

**Published:** 1899-02

**Authors:** 


ARTICLE VII.

### EXPOSING FRAUDULENT COLLEGES.

Report of the Committee on Foreign Relations of the National Association of Dental Faculties.

As to the irregular colleges, your committee considers it the imperative duty of this body to employ every possible means for their exposure and suppression. We believe that it should protect the good name of American dentistry and American educational institutions. In this faith your committee, through its chairman, authorized the expenditure of a reasonable amount of money in the prosecution of investigations of unrecognized and irregular schools, and secured the co-operation of a thoroughly competent man for this work. As a consequence, considerable progress has been made in the unearthing of some of them. But it will probably take years of persistent effort to accomplish all that is desirable. We have received the most encouraging letters from our confreres in Europe, and have been materially aided by some of them. We have been assured that if such work is continued it must result in the higher appreciation of this association in Europe, and in the perceptible raising of the estimation in which our degree is there held. Hence, we feel warranted in urging upon you increased zeal in the prosecution of the work already commenced.

We append the names of five Chicago institutions about whose status we have made inquiries and examination, and which we believe to be unworthy the patronage of dental students, and which are not recognized by reputable schools. We present the prospectuses of such as we have been able to induce to send them :

(1) "The Cosmopolitan Post-Graduate School of Dental Surgery." The name of the dean is published as C. A.

Weil, M. D. The President is a man by the name of Williams, a lawyer, with an office in the Unity Building. The Secretary is a druggist, by the name of Fred. Brunhoff. These gentlemen sign the diplomas, and the instruction given, so far as known, has been in the office of one dentist, Solomon, and it has been charged also at a well-known post-graduate school of Chicago for a month or so.

(2) "The German Medical College and German University of Chicago." This is located on West Thirteenth street, near Ogden avenue. The name of the dean is John Malok, M. D., who was formerly a barber in Berlin, but who came to this country and graduated from the Hahnemann Homœopathic Medical College. Malok is said to be all there is to this "college," and he offers to grant degrees in medicine, dentistry, philosophy, law, midwifery and divinity, or almost anything else.

(3) "The Independent Medical College." This is located at the corner of Van Buren and Leavitt streets, People's Institute Building. Thomas Armstrong, Ph.D., is the motorman. The post-office authorities have about run this man down, and have employed agents to procure bogus diplomas with the view to prosecution and suppression. J. H. Randall, Ph. D., M. D., (?) is the reputed Vice-President, and Charles M. Hovey, Secretary.

(4) "The German-American Dental College," located at 760 North Park avenue. Fritz W. Huxmann is the managing head of this school, which is duly incorporated. It professes to give professional instruction, and to be "regular," but it is not so acknowledged by other colleges. Huxmann was formerly connected with the Examining Board of the State, appointed by the too-well-known Altgeld, and he posed then as a pillar of the law. His diplomas are not recognized by our schools, nor are students received from his institution. He has advertised extensively abroad, and has been a source of bitter reproach to American dentistry. He is not here classed with the admittedly fraudulent operators, but it is thought that a considerable of investigation as to his methods and pretensions might be beneficial to dentistry.

(5) "Illinois Academy," 324 Burling street, Chicago, Ill. Prof. Dr. B. E. Winther, "Magnificus." This is a side issue and is presumably fraudulent. Sufficient data has not yet been procured concerning this affair. It should be completely unearthed.

The State of Illinois is a glaring example of this kind of vicious legislation, and nearly or quite all the fraudulent colleges are now located in the city of Chicago, to the great reproach of the State and the profession of dentistry within its borders. That city contains some of the very best of our professional educational institutions, and at the same time the most villainous imposters conceivable. Dentistry in Chicago can boast of as high-toned and eminent practitioners as are found anywhere in the world, and it is disgraced by some who appear to acknowledge none of the usually accepted professional obligations, while using the professional name to further their own illegitimate ends. Unfortunately it is sometimes hard for the uninitiated to tell them apart, for some of the latter have held responsible professional positions, and use that seeming endorsement in the pursuit of their illicit business.

Men unacquainted with professional educational affairs, who know not the wiles of designing tricksters who would take advantage of an innocent law to further their own selfish purposes, are not the best judges of what is proper legislation for the professions. In an unsuspecting moment, and without sufficient consideration, there was placed upon the Illinois statue books an enactment which, while assuming to further business interests, and honestly intended for their benefit, allows the incorporation under the law of associations that may carry on a fraudulent diploma business. So loosely or so nefariously drawn was this law, that for the merely nominal fee of registration, amounting to less than five dollars, totally unqualified men may be permitted to use diplomas ot qualification in the different professions. This seems a monstrous state of affairs, but it has been suffered to exist for years. The citizens of other States are powerless, for Illinois is supreme within her own jurisdiction, and she continues to protect her criminals in their villainy. The task of securing the repeal of this vicious law is too great for the courage of its reputable men, for ignorance and vice have struck hands in its maintenance. Even the excellent and influential Illinois State Dental Society has looked upon this condition with seeming indifference. As a consequence of the continuance of this demoralizing law, a considerable number of the practitioners of Chicago carry in their pockets, or exhibit on their walls, college charters conferring upon them the power to issue diplomas in dentistry. A number of advertising offices are legally conducted under such names as the "The Illinois Academy of Medicine and Dentistry," "The College of Painless Dentistry," "The Union College of Dentistry," etc., and the certificates of the Secretary of State, under the great seal of the State of Illinois, can be obtained certifying to their entire legal respectability and status. It seems to your committee that the decent part of the profession of this grand State should begin an agitation for the repeal of this vicious law. It is earnestly to be hoped that as soon as the professional men of the State are aroused from their lethargy and made to comprehend the enormity of the condition, they will present the subject before the legislature in its proper light, and the disgraceful law will be so amended that it will not apply to educational institutions, and the charters already issued under it will be very promptly canceled.

Some of the so-called dental colleges have no other existence than this State incorperation. They are owned and run by one man, and he perhaps sails under a false name. Of course, if they give no instruction, and yet confer degrees, they are amenable to the law against fraud. But their diplomas are not offered at all in this country, being only advertised abroad. They know very well that if they attempt to ply their trade at home they will speedily be brought to grief, and so they permit no proofs of their work to come to light in America. There is no indication of their business at their published address, and any letters sent to them from this country are carefully left unanswered. Their work is done through European agents. We can not locate them, and there are no proofs to be obtained in this country. Our confreres abroad complain bitterly of these swindlers, but they do not comprehend the situation, and when we ask them to obtain the proofs of their villainy, they reply that the miserable affairs are under our immediate notice, and we should get the testimony here.

Sometimes our professional journals, and some of our prominent men, and even professional organizations instituted for the purpose of regulating dental practice here, unwittingly further the objects of these men by falsely charging that respectable schools are practically engaged in the same business of granting irregular degrees, and thus they efface the line of distinction that the reputable colleges have been striving to set up. It is a singular fact that nearly or quite every application which approved colleges receive for irregular degrees comes from Europe, and because of these miserable villifications of respectable schools by American dentists acting with more zeal than discretion and more fervor than knowledge, there is not an American college that is free from these insulting applications.

This is the condition that confronts us in America. This association has done what it could, and advanced as fast as it could. It has been embarrassed by the lack of co-operation, and even by the active opposition of those to whom it had a right to look for help. It has been denounced because it has not taken the radical steps demanded by men who have little comprehension of the difficulties to be met, and who do not understand that the tone of the colleges, and the profession as a whole, can only be advanced by a movement that is made as a whole. At the most critical moment, the ground that has been gained has been lost through the absolute refusal of some of the colleges to vote to sustain the most moderate requirements, and it is astonishing that so much has been accomplished. This association has sharply drawn the line between the reputable and the disreputable schools, and despite the fact that over-zealous and unwise men have been industriously engaged in effacing it, and confusing the good with the bad by claiming that all have the same character, in this country the distinction is clear and well known. It should be, and if these ill-advised strictures are abandoned, it will soon be as well comprehended abroad. All that is necessary is to scan the list of the members of the National Association of Dental Faculties, and if the name of an institution granting a diploma is not found in it, that document is unacknowledged by this association. If any college that has a membership in this association grants a degree or accepts a student irregularly, the faith and honor of every other member is pledged to inflict the most condign punishment upon presentation of the proofs.

But it has been charged that violations of the rules have been committed by members without subsequent punishment. There appears to be an impression that it is the duty of the association to discipline a college upon mere rumors and to inflict punishment without proofs. This would be the rankest injustice. There has never yet been definite charges made against a college by any responsible party, without accompanying proofs or positive information where evidence could be found, without the most thorough investigation. It has been charged before your committee that the Stauber instance was such an one, But in that the implicated college corrected the error of its own volition. The remedy for infraction of our regulations thus rests in the hands of every respectable member of our profession, for so carefully has this association guarded this point that it has appointed a committee with plenary powers for the express purpose of investigating charges of irregularity brought between the sessions, thus offering swift as well as exact justice.—*Dental Brief.*

